# Unconscious Effects of Action on Perception

**DOI:** 10.3390/brainsci2020130

**Published:** 2012-04-16

**Authors:** Veronika Halász, Ross Cunnington

**Affiliations:** 1 Queensland Brain Institute, The University of Queensland, St Lucia, QLD 4072, Australia; E-Mail: r.cunnington@uq.edu.au; 2 School of Psychology, The University of Queensland, St Lucia, QLD 4072, Australia

**Keywords:** perception, action understanding, motor system, predictive coding

## Abstract

We spend much of our life predicting the future. This involves developing theories and making predictions about others’ intentions, goals and about the consequences of the actions we are observing. Adapting our actions and behaviours to the environment is required for achieving our goals, and to do this the motor system relies on input from sensory modalities. However, recent theories suggest that the link between motor and perceptual areas is bidirectional, and that predictions based on planned or intended actions can unconsciously influence and modify our perception. In the following review we describe current theories on the link between action and perception, and examine the ways in which the motor system can unconsciously alter our perception.

## 1. Introduction

Our actions and behaviours are continuously adjusted to correspond with changes in the environment and in social settings. To do this, the brain needs to rapidly and efficiently process incoming sensory information and match with predictions based on our current actions or intentions. Perception and action are therefore closely linked, and regulating the brain processes underlying perception helps us to achieve our goals in a constantly changing environment. Recent theories posit that information flows not just from perception to action, but also from action to perception, such that predictions based on our own actions or intentions can unconsciously influence our perception of others’ actions.

Our aim in this review is to explore how information from the motor system of the brain can unconsciously influence perception. Of course, perceptual guidance is crucial for our everyday actions, and there is extant literature on how sensory information links to the motor system for guiding our behaviour. However, very recent research has also begun to examine the inverse relationship—specifically, how actions can unconsciously influence perception. Mixed results are reported whereby actions can sometimes facilitate or attenuate our perception. Here we describe current theories on the link between voluntary action and perception, and examine the different ways that perception can be modulated by the motor system. We conclude by arguing that predictive models of action perception can best explain how our motor system unconsciously influences our perception.

There are three main theories that are used to explain how actions represented in the motor system link with perception—the *common coding theory*, the *direct matching hypothesis*, and *predictive models* of action understanding. These theories largely describe how we understand and perceive others’ actions, but can also describe how motor plans or intentions can influence perception.

These theories are all based on the fact that neural circuitry involved in action observation and perception overlaps extensively with those areas that important for executing our own actions. For example, during action observation, neuroimaging studies have demonstrated automatic activation of motor and premotor areas in the brain [1,2], while neurophysiological measurements show covert corticospinal motor pathway excitation [3]. Additionally, the link between action and perception also exists on a single-cell level. A subset of premotor and parietal neurons discharge when monkeys both execute certain actions and when they observe the same actions executed by others [4,5]. These neurons are called *mirror neurons*, while the phenomenon that observed actions elicits similar neural activity as executed actions can be collectively called *action mirroring*.

In reviewing these theories, an important distinction is whether they propose mechanisms for action understanding that are predominantly *postdictive* or *predictive*. Postdictive theories postulate that observers rely on motor memories or associations based on previous experiences in order to understand the observed actions. In other words, these theories suggest that the main task during action-observation is to decode sensory information to extract meaning after it is received in the brain, as if the system would ask the question: “What has just happened?” In contrast, predictive theories claim that, during action observation, our brain unconsciously makes predictions of the near future, already setting-up sensory processes for what we are most likely to perceive in the following instants, answering the question: “What will happen next?”. 

### 1.1. Common Coding Theory

**Figure 1:**
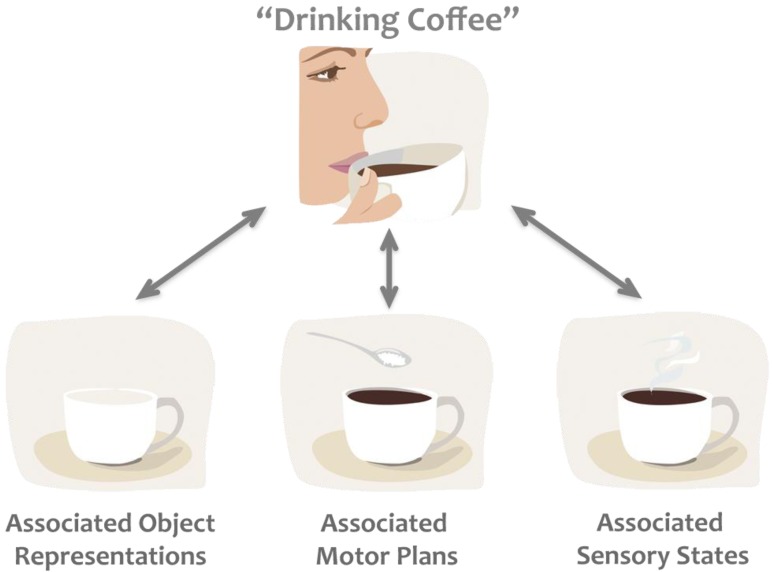
Example of the common coding theory. Thinking about “drinking coffee” activates associated codes, which frequently occur together, such as objects (e.g., coffee cup, coffee beans), motor plans (e.g., the way we like to hold our cup), and sensory states (e.g., the colour, smell, taste of coffee), biasing subsequent processing of any of these associated states.

The earliest theoretical framework on the connection between perception and action is the *ideomotor theory* [6]. It suggests that actions and internal images of actions are closely linked, and that actions are represented by their sensory consequences [7]. Building upon these basic ideas two widely cited theories have been proposed, first the *common coding theory* [8] and later the *theory of event coding* [9]. According to these theories, fundamentally the same areas of the brain are involved in perceiving and planning an event. For example, if we are about to have a cup of coffee, or maybe just smelling the coffee, the same areas of the brain become commonly active, as the motor acts and their associated sensory states are commonly coded in the brain (Figure 1). The common coding theory does not strictly define how information flows within this network. It is neither predictive nor postdictive for the same reason: while one thinking about “coffee” neural activity of past memories related to coffee drinking or future imagined events are equally likely to be activated. This indistinct nature makes the common coding theory flexible enough to explain several phenomena related to perception-action and action-perception connections. However, it is not exactly clear on how and why different codes become active or “remain silent” in any given situation; therefore it is hard to assess the validity of this theory scientifically. 

### 1.2. Direct Matching Hypothesis

**Figure 2:**
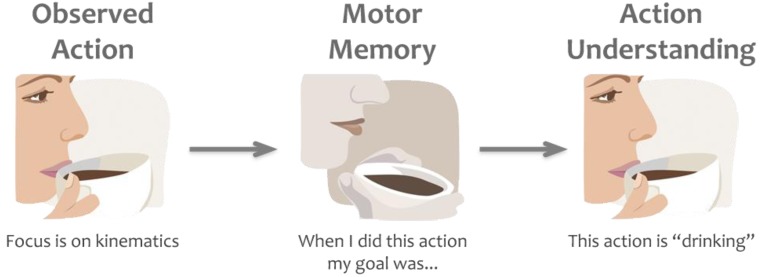
Example of the direct matching hypothesis. While observing a motor act we automatically map the kinematics of the observed action onto our own motor plans. By retrieving the goals and intentions (in this example “drinking”) behind those motor plans, based on our own prior experience, we understand others’ actions or goals.

One of the most popular theories that explain the function of action mirroring is the *direct-matching hypothesis* [10]. This theory claims that “an action is understood when its observation causes the motor system of the observer to ‘resonate’” ([10], p. 661). According to the direct-matching theory, action mirroring is a process of simulation that leads to understanding the goals of observed actions by automatically mapping those observed actions into the observer’s own motor system [11]. This is claimed to be a bottom-up or stimulus-driven process, whereby low-level representation of the observed movement kinematics triggers higher-level activation of the brain where goals and intentions are coded [12]. The direct matching hypothesis suggests a feed-forward flow of information whereby the visual information related to actions in occipito-temporal brain areas flows into the posterior parietal lobe and the premotor cortex (both of which contain mirror neurons) and leads to motor representation of the observed action for understanding of the action goals [4]. This classic view of the direct matching hypothesis is fundamentally postdictive; it suggests that observers project backwards in time to recover associated goals or intentions that they experienced previously while executing the same actions [13]. For example, while observing somebody picking up a cup, our brain matches the observed action with equivalent motor plans and identifies the goal of the action (e.g., “drinking” or “transporting a cup”) by activating the associated goals or intentions we have had previously when performing the same action ourselves (Figure 2). However, recent studies using single cell recordings also report mirror neurons that show activation related to action sequences that are about to happen [14,15]. These findings suggest that mirror neurons may support a more complex, predictive type mechanism for action understanding compared to the essentially postdictive process described by the classic direct matching hypothesis.

### 1.3. Predictive Models

**Figure 3:**
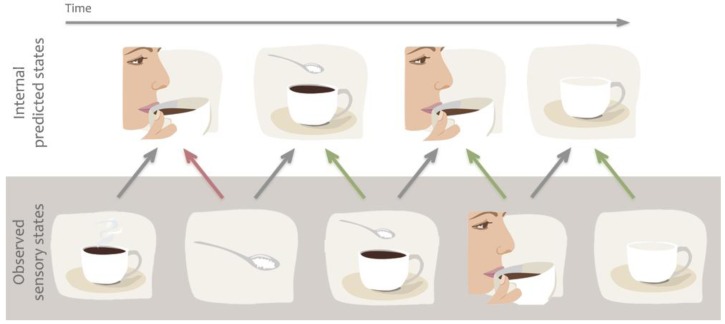
Example of predictive coding or forward models. We are constantly making predictions about the future state of our sensory system based on previous associations. Predictions are also quickly updated based on incoming sensory information to minimize prediction error. For example we predict that our friend will take sip from her coffee but when her hand grabs the sugar bowl we quickly alter our prediction.

A set of theories are essentially predictive in nature, and claim that action mirroring is used to predict actions, goals or sensory states that are about to occur, thereby readying our sensory systems for processing of the expected incoming sensory information [12,13,16,17]. While predictive models can also be described computationally from physical systems [18,19], in the present review we focus specifically on predictions of future states from internal models. These theories predominantly rely on the concept of internal *forward models* by which *emulators*, or mental simulations, provide a mechanism to estimate anticipated internal neural representations of external actions or events by real-time simulation of the consequences of those events [13,17]. For example, the *predictive coding* model of Kilner and colleagues [16] suggests that several forward and backward loops exist between the levels of a hierarchically organised system, and anatomical connections between these areas are reciprocal. The forward models suggest that, during action observation, we are constantly making predictions about the acting agent’s goals, intentions or next moves. These predictions then are fed-forward to influence the way sensory brain areas process information. For example, when we observe someone holding a coffee cup, the brain predicts the most likely outcome of the action, e.g., that the person takes a sip (Figure 3). However, if the person’s face suddenly changes and expresses negative emotions, the predicted outcome rapidly changes and the brain recomputes the next most likely outcome, e.g., perhaps to anticipate a comment from the drinker about the drink being too hot or having a bad taste. This type of prediction enables us to process expected sensory information or detect unexpected outcomes quickly and efficiently, and thereby to adapt our own behaviour to the environment or social settings. 

In summary, there are three main types of the theories on how actions and perception are linked. The common coding theory is the most widely used theory to explain how the motor system can influence perception. However, this theory does not define clearly the mechanism underlying how and why different representations become active during action observation. In contrast, the direct matching hypothesis describes a feed-forward nature of information flow during action observation, whereby low-level aspects of an action are matched to higher-level action representations and goals in order to understand others’ actions. This theory maintains that goals and intentions are extracted from observed actions in a mostly postdictive way. In contrast, predictive theories propose that the brain predicts the most likely future events, based on predictions about others’ goals and intentions. Forward models then translate these predictions of intentions to anticipated sensory representations that can influence the way we perceive the observed actions.

## 2. Actions Influencing Perception

The notion that information from the motor system can influence perception is in complete contrast with traditional views of brain organisation, in which sensory systems are considered the input end and motor systems the output end of the brain. These effects of actions on perception can be divided into two categories, one dealing with how long-term changes in the motor system with skill learning or motor dysfunction can effect perception, and the other focusing on real-time effects whereby our immediate motor plans or intentions can alter perception.

### 2.1. Effects of Long-Term Changes in the Motor System on Perception

#### 2.1.1. Motor Disorders

A crucial set of evidence regarding the effect of the motor system on perception comes from patient studies, in which dysfunction of the motor system also impairs action recognition. For example, stroke patients with a motor deficit affecting their contralesional upper limb not only show impairment in action recognition, but that impairment is significantly stronger when it corresponds to their hemiplegic arm [20]. Similarly, paraplegic patients with severe spinal injury are significantly impaired in detecting and discriminating the direction of biological motion in point-light walkers (animation sequences of human motion represented by the movement of the joints) compared with healthy individuals [21]. Deficits in motor planning have also been shown to impair the ability to discriminate the gestures of others [22]. Apraxic patients, who have impairment in performing complex movements following stroke, show a strong group-level correlation between motor impairment and the ability to recognise and perceive movements [23]; however, the authors point out that, at an individual level, intact motor production is not always necessary for action or object recognition [23]. Patients with motor impairment due to cerebellar lesions also show impairment in understanding the sequence of observed actions [24,25], suggesting that the cerebellum is heavily involved in sequencing executed and observed motor acts and probably also predicting the sensory consequences of both observed and executed actions (for a review see [26]). Finally, people with developmental disorders involving impaired movement performance also typically show impairment in biological motion perception [27,28,29,30]. These studies show that damage to the motor system impairs movement perception, implying that the perception of action relies on functioning of the motor system of the brain.

#### 2.1.2. Motor Expertise

Changes to the motor system with skill learning also affect perception. Whilst the term “expert eye” is often used colloquially, perceptual expertise is not hidden in the eyes, nor is it necessarily in our visual system. Several studies have shown how learning new motor skills or motor expertise changes the way we perceive observed actions [31,32,33]. An fMRI study measured brain activity in expert female and male dancers while they observed gender specific movements, such that visual exposure was equal for both types of movements but motor familiarity was gender specific for the participants [31]. Enhanced brain activity was found in shared action observation/execution areas of the brain while participants watched actions from their own motor repertoire. In a similar study, brain activity was correlated with the amount of physical practice in novel dance movements [32]. Furthermore, imitating artificial object movements also led to increased brain activity in perceptual areas [33]. The authors of these studies argued that, according to forward models, the specific motor knowledge of experts resulted in a quantitatively increased processing of observed actions, leading to a more precise prediction on how other’s actions unfold in time and space.

Indeed, studies have shown increased accuracy in discrimination tasks in motor experts. For example, active basketball players predicted the success of free shots more quickly and accurately compared to individuals with similar visual but less motor experience (sport journalists or coaches) [34]. Likewise, recognition of a gait pattern presented by point-light displays was higher after blindfolded training, with visual accuracy showing a strong correlation with the accuracy with which participants executed the learned movements [35]. These results indicate that increased visual accuracy does not originate from visual familiarity with the action, but from the expertise of the motor system.

Similar results have also been reported on accuracy in relation to sinusoidal movements [36], atypical movements novel for the motor system [37], or on dart throwing [38]. In the latter case, participants watched videos showing darts being thrown and were required to predict where the dart would land. Participants were significantly better at predicting their own throwing than other’s throws. Similarly, accuracy to identify point-light movements was highest when participants observed their own action, less precise but still above chance when friends executed the actions, but fell below chance level for strangers [39]. Changes to the motor system with skill learning or familiarity therefore appear to result in changes to perceptual abilities, supporting a critical role for the motor system in action perception.

Long-term motor practice not only increases visual accuracy but can also affect other perceptual systems. In an experiment by Repp and Knoblich [40], participants showed altered auditory processes as a consequence of concurrently performed actions. Participants heard ambiguous tone-pairs that could equally be perceived as rising or falling tones. Interestingly, when they made concurrent key-presses from left to right, in the direction of rising tones on a normal keyboard, they were more likely to perceive the sounds as a rising tone. Vice-versa, when they made right-to-left key-presses they more often reported hearing the tones as decreasing. Both pianists and non-pianists showed this effect, but it was significantly stronger for pianists. These results clearly show that the actions performed have an influence on how concurrent sensory stimuli are perceived, and importantly that extensive motor practise or skill can have an additive effect on this association between the motor system and perception.

### 2.2. Effects of Planned, Intended, or Executed Actions on Perception

#### 2.2.1. Facilitatory Effects

While studies of motor skill learning show long-term facilitatory effects of the motor system on perception, concurrently planned or executed actions can also immediately influence action perception. In line with results in the previous section, several studies have reported enhanced perceptual performance for stimuli that are congruent with concurrently planned or executed actions [41,42,43]. For example, in an experiment conducted by Lindemann and Bekkering [42], participants were prepared to turn an object clockwise or counterclockwise when presented with a “Go” signal that was also turning either congruently or incongruently with the planned action. Participants were faster to respond to the rotating visual cue and turn the object in the congruent condition than in the incongruent condition. The authors interpreted this result according to the common coding theory, whereby preparing to execute the action would have also prepared the visual system for perceiving the consequences of that intended action, hence resulting in faster detection times for congruent stimuli. Other studies have similarly reported faster reaction times when the prepared action and visual stimulus were congruent, and have been interpreted as a facilitatory effect of the action on perception [41,43]. These types of studies, however, are open for alternative interpretation, as it is possible that the presented stimulus affected action execution, particularly when it was incongruent, and not that the prepared action facilitated the processing of the visual stimulus.

#### 2.2.2. Twisted Illusions

A clever way to measure changes in perception is to use ambiguous sensory stimuli and perceptual illusions, as did Repp and Knoblich [40] with ambiguous tones. In a study by Wohlschläger [44], the actions performed by participants determined how they perceived the direction of motion of an ambiguously rotating sphere. In their study, participants watched a rotating sphere that could equally be perceived as turning clockwise or anti-clockwise. When participants concurrently turned a knob either clock-wise or anti-clock-wise, they were more likely to perceive the sphere to be rotating in the same direction as their action; that is, their planned actions were shown to prime the perceived direction of the visual stimulus. Moreover, their study also showed that the priming effect was present when the goal of the planned action shared a common dimension with the visual display and that a strict correspondence between the actual hand movement and the visual motion display was not necessary for the effect to be observed. The effect of actions on perception can rely on higher order action representations, such as action goals, and a strict matching between the perceived stimulus and low-level kinematics is not crucial for the modulation effect. This study is one of the few that take into account the hierarchical organisation of the motor system, and addresses the possible effects of this hierarchy on the action-perception link.

Interestingly, only actions that are dependent on the currently perceived stimulus influence the perception of that stimulus [45]. In a recent study, participants were asked to report the direction of an ambiguously turning stimulus again, but either by turning a manipulandum or making key presses. When indicating the perceived direction by rotating the manipulandum, incongruency between the perceived and reported direction destabilized the percept, while congruency stabilized it. However, this effect disappeared when participants reported the perceived direction by key presses, even if they were concurrently performing a predefined (congruent or incongruent) turning movement on the manipulandum [45].

**Figure 4:**
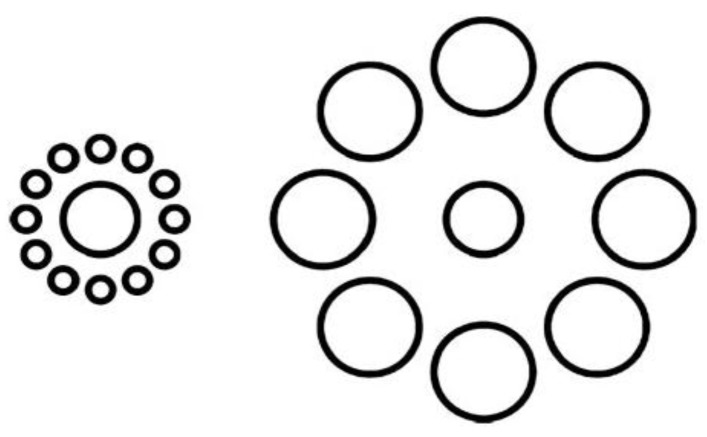
The Ebbinghaus illusion. This illusion leads to the misperception of the size of the central circle; however the effect decreases significantly if there is a grasping or pointing action directed to the central circle.

Another experiment involving visual illusions showed how motor plans can reduce visual illusory effects. The Ebbinghaus illusion (Figure 4) is a classic visual illusion in which the central circle surrounded by small circles appears considerably larger than the circle surrounded by large circles. Vishton *et al*. [46] showed that if participants were asked to grasp or touch the circle they perceived larger, rather than verbally reporting which was the larger central circle, the magnitude of the illusion was significantly decreased. This suggests that motor plans can partially correct for deceived perception in visual illusions. In summary, the above experiments on visual illusions indicate that the motor system can unconsciously alter sensory ambiguity to be in line with concurrent motor plans.

#### 2.2.3. Action-Affected Blindness

In striking contrast to the above results, other studies report an attenuating or inhibitory effect on the processing of visual stimuli that are congruent with actions. Müsseler and Hommel first described an apparent blindness to response-compatible visual stimuli, calling it *action-affected blindness* [47]. This effect later was replicated by several studies, all using a very similar method [48,49,50,51,52]. Participants plan left or right keypresses, but, just before they execute the action, an arrow is presented very briefly and they must report whether the arrow is pointing left or right. The perception of this arrow is impaired if it is pointing in the same direction as the planned action. The interpretation of this effect by the common coding theory suggests that, since planned and perceived actions share a common encoding, the planned action establishes the code associated with its execution and sensory consequences. Subsequently, when the congruent visual stimulus appears, this code is already represented and is less accessible for perception, and thus the perception of the congruent stimulus is impaired [48].

With an interesting twist on the original experiment, Stevanovski and colleagues revealed that this blindness effect does not rely on low-level congruency between the presented stimuli, but on higher-level representations [50]. In their experiment, participants were instructed that arrowhead symbols (*i.e*., “<” or “>”) were actually headlights, so that the direction in which they were pointing was reversed compared to the original experiment. The blindness effect was still present, but now in the reversed direction. Thus, this experiment indicates that the action-blindness effect is not due to low-level similarity, but depends on how we interpret the stimulus in a given context.

Motor plans have also been shown to cause longer-lasting inhibitory effects on action perception. Cattaneo and colleagues [53] showed that, after a training session of simple pulling or pushing movements, participants were more prone to perceive movement of an ambiguous stimulus in the opposite direction compared to that trained in the motor task. The authors explained these results by postulating that mirror neurons, linking actions to perception, showed an adaptation effect as the consequence of motor training. This effect then was carried over for the visual perception task, resulting in decreased sensitivity in those neurons that equally encode executed and observed actions. In summary, the above studies suggest that motor plans can decrease the sensitivity of visual perception for stimuli that are congruent with the current or recently executed motor plan.

#### 2.2.4. When Similar Repels and Different Attracts

Most of us probably spent time as a child trying to push together the like poles of bar magnets, contrasting with the strength of the attracting force between north and south poles. The same effects are hypothesised to occur in perception and concurrently executed actions: similar repels and different attracts. When there is congruency between the motor plan and the sensory information, the latter gets “repelled”, or in other terms does not reach consciousness. On the other hand, if there is a mismatch between the motor plan and the sensory information we are quicker and more precise to perceive that information as it would attract our attention.

For example, Zwickel and colleagues asked participants to make hand movements in a certain direction while simultaneously reporting the direction of the motion of an independent stimulus [54]. Motion deviations of this independent stimulus were detected faster when their direction became incongruent with the executed hand movement. Similar effects have been reported for visual discrimination of hand movements [55], judging weights [56], or judging gait speed [57]. Zwickel and colleagues interpreted these results based on the common coding theory, and argued that people are more sensitive to perceive stimuli that deviate from the anticipated effects of their actions [54]. When an observed stimulus matches the expected sensory consequences of the planned action, consciously perceiving it is less important because it does not carry any additional information in assisting the effective execution of the action. However, when the observed stimulus contradicts the expected sensory outcomes of the action, rapid perception of that stimulus can be crucial for modifying our action to better fit the environment to achieve our goals.

The above argument can also explain the results of Bortoletto and colleagues who showed that motor plans can influence early visual processing of an observed action [58]. The authors measured visual event-related potentials related to the perception of hand actions while participants were planning either congruent or incongruent hand actions [58]. Two components of early visual processing, namely N170 and Vertex Positive Potential were significantly higher to observed actions when those actions were incongruent with the planned actions. This result indicates that even early visual processing of observed actions—that is out of conscious perception—can be modified by motor plans. 

A curious case that fits this section is related to an everyday experience with which we should be all familiar: why we cannot tickle ourselves. A touch feels ticklish when it is somewhat unpredictable; when we are concentrating on a very obvious movement we might not feel the tickle at all. Of course when we decide to move, our brain has a very precise prediction of what we are going to do and by predicting the sensory consequences of those movements we become less sensitive to perceive them. Blakemore and colleagues tested this theory by inducing delays and variation to participants’ movements when they were intending to tickle themselves [59]. When the sensory stimulus and the planned action were more consistent, the less ticklish the touch felt; however, the more inconsistent they were, the more ticklish the touch became. In conclusion, a complex two-fold interaction between perception and action exists. Motor plans can reduce the sensitivity for perception of congruent sensory information, but can also enhance the perception of a stimulus that is incongruent with concurrent actions.

#### 2.2.5. Dynamic Systems, Complex Interactions

Having previously highlighted the key findings related to action modulated perception it is important to stress the complexity of the brain processes underlying these phenomena. While experimental studies usually reduce tasks to a single motor plan and a sensory stimulus, in the real world there is dynamic, continuous interplay between action and perception. At present there is a relative lack of research investigating more real-life interactions between action and perception. One such study by Bhalla and Proffitt [60] showed that hills appear steeper to people who are wearing a heavy backpack, fatigued or perceive their physical fitness as relatively low. Based on forward models the effect can be explained as follows: the motor plan forms the base of a predicted sensory state; this prediction is influenced by the relative heaviness of the backpack in a way that the predicted execution of the motor plan seems more tiring; this prediction in turn can influence the perceived steepness of the hill in correlation with the predicted difficulty of the task. Similarly, objects look closer when a tool is held and the intention is to touch the object with the tool than when the tool is not held or there is no intention to touch the object [61]. These examples demonstrate how brain processes during action execution and observation comprise a complex dynamic system in which there is a constant process to interpret, suppress or enhance sensory information based on our motor plans and goals. Information during action observation and action execution flows to and from the sensory areas of the brain and intricate interactions and modulatory factors, relating to our action goals and intentions, influence what we perceive about the world.

## 3. The Case for Predictive Models

There is ample evidence in the recent literature suggesting that the motor system not only receives information from the sensory areas of the brain, but also influences sensory processing and thereby unconsciously modulates our perception. The effect that actions can have on perception can be divided into two types: effects of long term motor expertise on subsequent perception, and immediate effects of either planned or executed actions on perception of concurrently observed stimuli. While studies of motor expertise show long-term facilitatory effects of the motor system on perception, planned or executed actions have been reported to either facilitate or attenuate visual perception of the concurrent stimulus. In the following we will review how the key theories on action observation relate to this modulatory effect and we will argue that predictive or forward models can best explain the complex interactions between motor and perceptual systems.

The common coding theory proposes that repeatedly paired actions and sensory outcomes may strengthen common codes or representations and lead to facilitation of perceptual performance during associated actions. Simultaneous activation of perceived sensory information and anticipatory effects of actions, however, can also lead to interference effects, thereby resulting in decreased perceptual performance. This decrement is suggested to arise because the commonly-coded action representations are already occupied from action planning and less sensitive to new sensory or perceptual information. The common coding theory, however, is only a theoretical framework and does not define clearly *why* concurrently performed actions sometime facilitate or attenuate perception. The direct matching hypothesis, on the other hand, proposes that low-level visual information about observed actions are mapped to the observer’s motor system where, through action mirroring, the goal of the action or intention of the actor is decoded. This theory is essentially postdictive in nature, emphasising information flow only from visual to higher cognitive areas, making it difficult to explain how actions may have a feed-forward effect on perception.

In contrast, we argue that predictive or forward models are the best candidates to explain the variety of effects that the motor system can have on perception. These theories propose that planned actions or predictions of observed actions lead to anticipated sensory representations of the outcomes of the action [12,13,16,17]. Forward models explicitly propose information flow from motor to visual areas and can therefore explain how and why perception may be modulated by motor plans or intentions.

According to predictive models, whenever we are preparing for an action or watching others move our brain makes predictions about what we are going to see, hear, and feel in the following instants. We are also automatically and unconsciously making predictions about what are other people’s goals and intentions based on their actions. There is a constant information flow between higher-level cognitive areas of the brain, the motor system, and the sensory system that enables us to predict anticipated actions and expected sensory consequences of those actions. When we gain expertise in some motor acts we become more efficient at predicting the sensory consequences of those actions [34,35,38]. Similarly, our predictions of other’s actions are more precise the more familiar we are with the acting person [39].

The picture becomes more complex, however, if we focus on the immediate effects of planned or executed actions on perception. When we act, or prepare to act, a sensory prediction based on our motor plan is generated and used to match or compare with incoming sensory information during perception. If this external sensory information is ambiguous it is affected by the sensory prediction based on our actions. This logic can explain how an ambiguous tone pair is perceived as rising or falling depending on whether our actions involve moving left to right or right to left [40], or create the illusion of a stimulus turning clockwise when our own hand actions involve turning clockwise at the same time [34,44].

There are also times when there is incongruency between what we perceive and what our motor system predicts. For example, in the case of the Ebbinghaus illusion (Figure 4) what we see is different from what our motor system predicts. When we move towards an object, we automatically and unconsciously adjust our fingers to take up a position that allows the best manipulation of the object [62]. This prediction based on our action can then weaken the perceptual bias of the visual illusion. Crucially, this effect only exists when we are planning to act upon the object, as passive observation does not involve any activation of motor plans [63].

When motor plans and the perceived stimulus overlap, experiments often report action-blindness effects [47,48,49,50,51,52]; that is, the relative blindness or missed perception of a stimulus that is congruent with the action-plan. Under limited resources, the system should filter out unnecessary information and focus on detecting stimuli that do not match predictions and therefore may require modification of actions. A stimulus that fits the predictions of the ongoing motor plan requires no special attention, and is thus less likely to reach consciousness in comparison to a stimulus that differs from the predicted sensory states.

The above logic can also explain the repellent effect of actions on concurrently perceived stimuli. The predicted sensory state, based on the motor plan, can influence or alter the perceived sensory information, resulting in a relative insensitivity for congruent stimuli [54,55,56,57,59]. However, sensory information that is incongruent with the predicted state is processed rapidly [58] as it is most likely to carry information important to modify our motor plans. Table 1 illustrates how the relationship between sensory information and concurrent motor plans may manifest in unconscious effects on perception.

**Table 1:** Relationship between sensory information and concurrent motor plans and their consequent perceptual effects.

Sensory InformationCompared to Motor Plan	Reported Perceptual Effect
Same	sensory information does not reach consciousness “action-blindness effect”
Similar	slow detection of stimuli, somewhat biased towards motor plan
Ambiguous	perception biased towards the direction of the motor plan
Different	quick detection of stimuli, no bias towards motor plans

Whatever theory is used to interpret effects of actions on perception, there is one common aspect that seems to be central and future studies might like to address: that is, congruency between the perceptual stimulus and the action. The action observation network is thought to be organised in a similar hierarchical manner to the motor system. Actions are formed by a sequence of steps, and these steps are organised hierarchically ([64]; or for a review see [65]). At the top of the hierarchy is an overarching goal, which needs to be achieved by completing sub-actions. These sub-actions are built from co-ordinated motor movements that, in turn, are built from individual muscle activations. It is hypothesized that the action observation network has a similar hierarchical organization in which different aspects of actions (e.g., goals, kinematics) are represented at different neural levels [66]. To date, however, it has remained unclear how congruency between the motor and perceptual system interacts to facilitate or attenuate perception of the stimulus. Most researchers fail to define the specific dimensions along which congruency may be varied, and so it is still unclear which level of action representation leads to effects of the motor system on perception. Furthermore, future studies should investigate the temporal and spatial dynamics of the modulating effects of actions on perception. The timing of the perceived stimulus compared to the action (*i.e*., whether it appears during the planning phase of the action or during execution) should be an important determining factor of how motor and visual systems interact in the brain.

More studies are needed to clarify how action and perception are linked in clinical populations. As described above there is a strong correlation between motor deficits and perceptual sensitivity in some clinical groups, but symptoms can be highly variable between individuals. Better understanding of how information flows between motor and perceptual areas would help to develop specific treatments for stroke patients or patients with severe motor disorders, such as cerebral palsy. Furthermore, it is still unclear in the developmental disorders literature whether an initial motor deficit leads to decreased perceptual sensitivity or the correlation is the result of complex interaction within the action-perception network (for an interesting paper on this issue see [67]).

## 4. Conclusion

In this review we have highlighted some of the existing literature on how our visual perception is unconsciously influenced by plans for action encoded in the motor system and reviewed the main theories applied to describe the action-perception link. Predictive theories, suggesting the existence of internal forward models and emulators, could best explain how information from the motor system can modulate perception. These theories claim that, during action observation and execution, we are constantly making predictions about the future and representing expected states in our sensory system. These predictions then modulate the way our brain processes the incoming sensory information to influence what we perceive. In this sense, action-modulated perception sheds light upon one of the core but silent mechanism of our brain: how we predict the future and how those predictions influence what we perceive and understand of the world around us.
